# Editorial: Growing Up in a Digital World - Social and Cognitive Implications

**DOI:** 10.3389/fpsyg.2021.745788

**Published:** 2021-09-30

**Authors:** Mikael Heimann, Adriana Bus, Rachel Barr

**Affiliations:** ^1^Department of Behavioral Sciences and Learning, Linköping University, Linköping, Sweden; ^2^Faculty of Arts and Education, University of Stavanger, Stavanger, Norway; ^3^Department of Education, Eötvös Loránd University, Budapest, Hungary; ^4^Department of Psychology, Georgetown University, Washington, DC, United States

**Keywords:** early childhood, digital media, learning, language, book reading, joint media engagement, technoference, robotics

Digital media availability has surged over the past decade. Most of us regularly check our emails, video chat, follow social media, search for new information, and play games. We frequently swap the real world for the digital world. It is the new “normal!” Children growing up today use digital media for learning and entertainment and to make social connections. The increasing usage of digital media has caused grave concern among parents and teachers. Rapid growth in access has been accompanied by similarly rapid growth in research on the effect of digital media. A search conducted in early July 2021 that included four major databases—Scopus, PubMed, PsycInfo, and ERIC—returned 1,777 hits when combining the search terms “digital media” and “screen time” with the age specifiers “infancy” and “preschool” (see Figure 1). A vast majority of the identified output, 1,269 hits, is from publications dated January 2016 to December 2020. Phrased differently, the mean average number of publications per year was 0 during the 1990s, 13 during the first decade of the twenty first century, and 176 from 2011 to the end of 2020. However, these publications often failed to consider the family context and socio-cognitive implications of digital media. As a result, there are many unanswered questions such as: What role do factors like content, context, and culture play in determining the impact of digital media, for good or for ill, on children's learning and development? The current Research Topic aims to tackle some of these questions.

**Figure 1:**
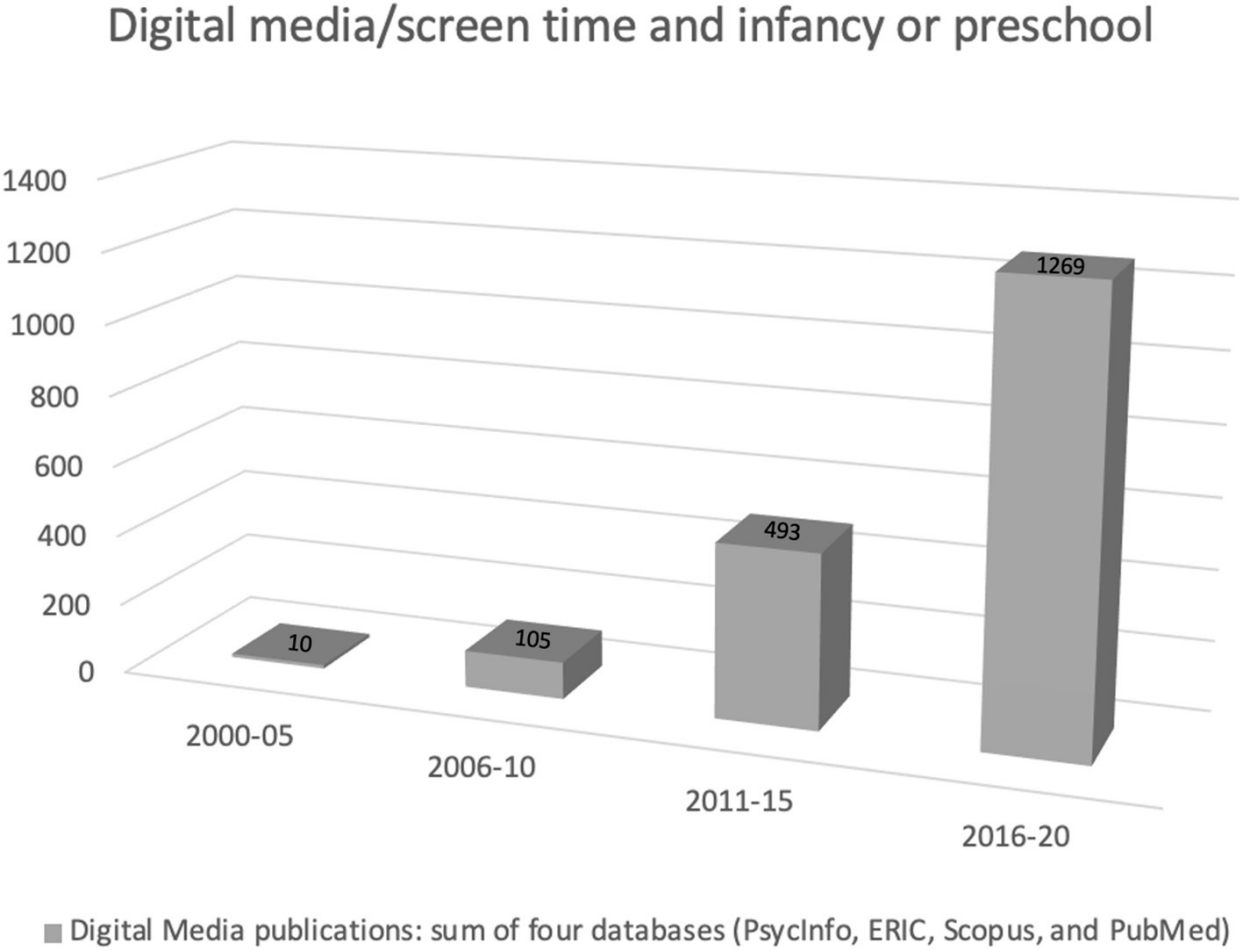
Returned hits from a search combining the terms “digital media” and “screen time” with “preschool” or “infancy.” Search date July 3, 2021.

The book includes 18 papers organized into three sections, one that focuses on book reading and language, one that covers potential risks associated with early media use, and one group of studies brought together under an umbrella we call New Developments. Some papers cut across sections and could have been included in more than one section. We are pleased to report that a majority of the papers result from international collaborations representing work conducted in nine countries. Six papers are from North America (Canada and USA), 10 from Europe (Germany, Italy, Norway, Sweden, and United Kingdom), and two from Asia (Israel and Singapore).

## Book Reading and Language

Digital media lends itself to storytelling, leading to an expansion in the ways children encounter stories. Apart from oral and traditional print books, even the youngest children have unprecedented access to film, apps, and games. The result is that most young children come across stories in formats other than traditional paper books. Therefore, it is not surprising that quite a few contributions focused on how these newly formatted stories relate to traditional book reading.

The current set of studies targets several sub-questions inherent to the new ways of encountering stories. The Courage et al. study tests whether 2- and 3-year-olds can operate a tablet purposefully to achieve a goal and, for instance, learn new information from a picture book app compared to a matched paper book. Others focus on the impact the digital book has on adult guidance. Müller-Brauers et al. zoom in on the narrative potential of a commercial digital picture book app and found that despite the helpful narrative animations provided by the app, most parents failed to fully exploit the narrative potential. In the same vein, Hoel et al. explore how early childhood educators prepare young children to participate in a shared digital-book reading session before the session and how successful they are in using typical features of digital books.

Crawshaw et al. explore a new storytelling technique, the film-like format, and how that contributes to story comprehension; to this end, they compare what children retain from a story after sharing a wordless picture book with the parent or watching a video of the same wordless story. Gaudreau et al. wonder how vital the physical presence of the adult is for comprehending a picture book. They compare the effects of a prerecorded pseudo-contingent condition with a video chat or live condition and report that 4-year-old children can comprehend a book equally well when read over video chat than when presented live.

## Potential Risks

Contributors examined how the content and context of media exposure were associated with decreases in the quality of play and language interactions, sleep, and focused attention. Two short-term longitudinal studies by Gueron-Sela and Gordon-Hacker and McHarg et al. examined multiple dimensions of media exposure that predicted later poorer attention and executive functioning outcomes. The use of longitudinal designs and the more detailed media exposure measures are important current directions.

Three studies used the CAFE media assessment questionnaire, which is part of the CAFE set of tools described by Barr et al. In Italy, Bellagamba et al. found that Italian children were exposed to media at similar levels to English speaking children from the US and the UK. Higher levels of media exposure were associated with poorer sleep habits. In Sweden, Sundqvist et al. examined how a 2-year-old's language use across the day is associated with daily media use. More direct exposure to media without active parent involvement was associated with poorer language outcomes. However, joint media engagement and book reading were associated positively with language. In Germany, Konrad et al. found that parental quality decreased when parents received a message on their phone during a free play session. Some parents also completed a paper version of the questionnaire and the change in interactional quality was the same suggesting that texting may be similar to other everyday interruptions. These findings suggest that complex patterns of media usage are associated with several domains.

## New Developments in Digital Media Research

This broad heading does not imply absolute uniqueness, but it is our view that these papers represent new and evolving subfields. Sun and Yin discuss how variation in input affects bilingual children's language learning. For bilingual children in Singapore, multimedia resources are more important for Mandarin learning than for English. This finding is explained by an unbalanced bilingual environment that provides poorer input for Mandarin learning than for English.

How do children evaluate information from different types of digital media? Hassinger-Das et al. studied this in a group of 117 children aged 3- to 8-years. YouTube videos are more attractive than smartphone or TV videos. This occurred despite the finding that the children tended to believe the YouTube information to a lesser degree.

Three studies focus on new aspects of co-media use. First, in an innovative study, Dore et al. analyses non-linear dynamics of how joint media engagement (JME) affects language development in 6- to 8-year-old children. Surprisingly, it is not until the number of hours children spend with digital stories (films, games, apps) exceeds 5 h per day that new media have a demonstrably negative impact on language development. Their findings pave the way for a more nuanced perspective on the effect of digital media in young children.

Low JME seems to be especially detrimental for children with high media use. In an experimental study of 2-year-olds, Heimann et al. report that JME did support learning from 2D media although not to the level of a 3D presentation. Finally, Ochoa and Reich show the influence of income and education in an interview study of Latin families. Parents graduated from high school stress the importance of co-using media but not parents with lesser education.

A different and new aspect of how children are affected by digital information is presented by Tolksdorf et al. who compared 4–5-year-old children's social interaction with a social robot and a human person. The children used social referencing in both interactions but significantly more so when interacting with the robot.

## Future Directions and Theoretical Implications

In sum, the papers demonstrate both the potential risks and benefits of early media exposure. If the content and context are right, digital media might provide a rich window to learning in new and exciting ways; to explore the world and social connections. Studies on the role of JME suggest promising avenues in which to work with families to use media effectively. The content also matters. Books, for instance, take new exciting formats due to technology and new storytelling techniques may open up opportunities to enjoy and comprehend stories.

Due to rapid technological advances, however, there remain several gaps in the literature. For example, modern media are mobile, interactive, and often short in duration, making them difficult to remember when parents, teachers, relatives, or older children respond to questions about media use. Although standardized measures of media usage are still being developed, it was encouraging that many of the included studies used more comprehensive multi-dimensional exposure measure. But researchers should also move beyond the exclusive use of parent reports and integrate direct observation of behavioral, physiological, or neural responses and use longitudinal approaches to capture the trajectory of exposure patterns.

Although we were able to solicit manuscripts from multiple countries, the samples recruited for the Research Topic were still WEIRD. Thus, we need to know more about cultural variation and for whom does media work. Notably, only Ochoa and Reich and Sun and Yin directly examined cultural implications. Future research should consider how patterns of media use are similar and differ between countries as a function of different parenting practices and include detailed multiple-dimensional media measurement.

Digital media provides exciting new opportunities for learning that have not been fully explored. In the current Research Topic, researchers examined different approaches to storytelling and social interactions. However, most research is based on standard materials and does not experiment with new technology-enabled possibilities. For instance, most contributions to this collection of papers targeting book reading do not control the enhancements in the target books but use what the commercial market offers. The fact that commercial design is more or less accidental may partly explain why findings are often inconsistent and hard to interpret. Digital book reading research will improve if researchers use materials grounded on conceptual frameworks. For example, Kucirkova and Littleton attempt to advance the digital-book format by theorizing about the distance between the familiar and the novel words of the story and propose to narrow the gap between reality and the interpretations of reality by adding other senses (e.g., taste and smell).

Instead of materials available on the commercial market, it might be essential to create materials that align a conceptual framework. None of the studies produced new technology to explore the hidden potential of technology. Research grounded in multimedia learning that tests how the format optimally benefits young children's story comprehension and incidental word learning is sorely needed. To achieve that goal, we need new collaborations between app developers, computer specialists, literacy educators, and specialists in digital learning, which seem indispensable to forward our insights on effective use of technology during early childhood.

Finally, we hope that the collection of papers will serve as a window to our current state of knowledge, inspire new researchers to enter the field, and motivate new collaborations among those already active.

## Author Contributions

MH, AB, and RB contributed equally to the writing of the Editorial and all authors approved the submitted version.

## Funding

This research was in part supported by grants from the Swedish Research Council for Health, Working Life and Welfare (2016-00048) to MH.

## Conflict of Interest

The authors declare that the research was conducted in the absence of any commercial or financial relationships that could be construed as a potential conflict of interest.

## Publisher's Note

All claims expressed in this article are solely those of the authors and do not necessarily represent those of their affiliated organizations, or those of the publisher, the editors and the reviewers. Any product that may be evaluated in this article, or claim that may be made by its manufacturer, is not guaranteed or endorsed by the publisher.

